# Fallopian Tube Cytology for Exploratory Detection of Adnexal Malignancy: Prospective Evaluation of the CytoSaLPs Score in an Ex Vivo Surgical Cohort

**DOI:** 10.3390/cancers18172868

**Published:** 2026-09-04

**Authors:** Victoria Psomiadou, Sofia Lekka, Theodoros Panoskaltsis, Abraham Pouliakis, Eleni Tsouma, Natasa Novkovic, Helen J. Trihia, Olympia Tzaida, Dimitrios Korfias, Panagiotis Giannakas, Christos Iavazzo, Christos Papadimitriou, Nikolaos Vlahos, George Vorgias

**Affiliations:** 1 Gynecological Oncology Department, “Metaxa” Cancer Hospital, 18537 Piraeus, Greecevorgiasgeorge@yahoo.gr (G.V.); 2 Gynaecological Oncology Unit, 2nd Academic Department Obstetrics & Gynaecology, Aretaieion University Hospital, 11528 Athina, Greece; 3 Second Department of Pathology, “Attikon” University Hospital, Medical School, National and Kapodistrian University of Athens, 12461 Athens, Greece; apouliak@med.uoa.gr; 4 Cytopathology Laboratory, “Metaxa” Cancer Hospital, 18537 Piraeus, Greece; 5 Department of Pathology, “Metaxa” Cancer Hospital, 18537 Piraeus, Greece; 6 Oncology Unit, 2nd Department of Surgery Aretaieion Hospital, National and Kapodistrian University of Athens, 11528 Athens, Greece

**Keywords:** CytoSaLPs score, fallopian tube cytology, ovarian cancer early diagnosis, adnexal malignancy, tubo-ovarian carcinoma

## Abstract

**Objective:** Ovarian, fallopian tube, and primary peritoneal cancers remain among the deadliest gynecological malignancies, largely because most cases are diagnosed at an advanced stage and no effective screening strategy is currently available. Increasing evidence suggests that many high-grade serous ovarian carcinomas originate from the fallopian tube. We aimed to explore the diagnostic performance of ex vivo fallopian tube cytology and the CytoSaLPs score for detecting tubal and adnexal malignancies in women undergoing salpingectomy or salpingo-oophorectomy. **Methods:** We conducted a prospective single-center observational study including 304 women undergoing salpingectomy or salpingo-oophorectomy for benign, premalignant or malignant gynecological indications between 2020 and 2023. Ex vivo cytological brushing of the distal fallopian tube was performed before fixation, followed by histopathological examination using the SEE-FIM protocol where appropriate. The primary analysis was performed at the specimen level. Of 544 paired specimens initially available for cytology–histology correlation, 53 non-diagnostic cytological specimens were excluded from the primary diagnostic performance analysis, leaving 491 evaluable paired specimens. Fallopian tube cytological findings were compared with histopathology as the reference standard. The discriminatory ability of the CytoSaLPs score was explored using receiver operating characteristic analysis. **Results:** Fallopian tube cytology demonstrated high sensitivity for histologically confirmed tubal malignancy, although specificity was moderate. Based on the primary specimen-level analysis, sensitivity was 94.4% and specificity was 71.0%. When fallopian tube cytology was compared with ovarian histology, sensitivity was 72.9% and specificity was 72.4%. For the adnexa considered as a single anatomical entity, sensitivity was 76.5% and specificity was 70.7%. The CytoSaLPs score showed good discriminatory ability for fallopian tube malignancy (AUC 0.8534), moderate discrimination for ovarian malignancy (AUC 0.6790), and fair discrimination for adnexal malignancy (AUC 0.730). The optimal score thresholds were derived from the same dataset and should therefore be considered provisional. Three serous tubal intraepithelial carcinoma lesions were identified histologically; two showed cytological abnormalities and elevated CytoSaLPs scores, whereas one specimen was non-diagnostic. **Conclusions:** This exploratory proof-of-concept study suggests that ex vivo fallopian tube and the CytoSaLPs score may provide a structured approach for detecting cytological abnormalities associated with tubal and adnexal malignancy. However, the findings were obtained in a tertiary gynecologic oncology population under ex vivo conditions, non-diagnostic specimens occurred in approximately 10% of samples, and the scoring system was developed and evaluated within the same cohort. Independent external validation and evaluation using clinically applicable in vivo sampling methods are required before any clinical implementation can be considered.

## Simple Summary

High-grade serous carcinoma is increasingly believed to originate from the distal fallopian tube epithelium, yet no effective screening strategies currently exist for ovarian or other adnexal malignancies. Previous studies investigating fallopian tube cytology have been limited by small sample sizes and the absence of standardized cytological scoring systems. In this prospective exploratory cohort, ex vivo fallopian tube cytology demonstrated high sensitivity for histologically confirmed tubal malignancy. The CytoSaLPs score provided a structured framework for evaluating cytological abnormalities and demonstrated good discriminatory ability for tubal malignancy. These findings support further investigation of fallopian tube cytology, particularly in combination with molecular biomarkers. However, the method remains investigational and is not suitable for routine screening or clinical decision-making without external validation and development of reproducible in vivo sampling approaches.

## 1. Introduction

Epithelial ovarian cancer (EOC), primary fallopian tube carcinoma and primary peritoneal carcinoma comprise a heterogeneous group of gynecological malignancies associated with substantial morbidity and mortality. Despite advances in surgical techniques and systemic therapies, overall survival remains limited, largely because most cases are diagnosed at an advanced stage. These malignancies account for approximately 2.5–3.1% of all cancer diagnoses and remain among the leading causes of gynecological cancer-related mortality worldwide [1].

Epithelial tumors account for approximately 90% of ovarian malignancies [2]. Ovarian and related pelvic carcinomas comprise several distinct histological and molecular subtypes with different biological pathways. Type I tumors generally develop through relatively slow progression from precursor lesions, whereas type II tumors are typically high-grade and aggressive with high-grade serous carcinoma, representing the most common subtype [3].

Increasing evidence indicates that a substantial proportion of high-grade serous carcinomas arise from the distal fallopian tube, particularly the fimbrial epithelium.

Serous tubal intraepithelial carcinoma (STIC) has been proposed as a precursor lesion for many high-grade serous pelvic malignancies [4,5]. Kurman et al. further suggested that low-grade serous ovarian tumors may also originate from fallopian tube lesions [6]. Fallopian tube–ovarian malignancies are occasionally detected through abnormal cervical cytology, with atypical glandular cells prompting evaluation for upper genital tract lesions [7]. This shift in the understanding of pelvic serous carcinogenesis has generated interest in the fallopian tube as a potential target for early detection and prevention strategies.

Nevertheless, despite extensive investigation, no screening strategy has been shown to reduce mortality from ovarian cancer in the general population. Large randomized trials evaluating CA125-based algorithms and transvaginal ultrasonography have not demonstrated sufficient benefit for population-based screening. There is therefore an ongoing need for novel approaches capable of identifying early malignant or precursor in the tubo-ovarian epithelium.

The CytoSaLPs score was previously proposed as a preliminary cytomorphological framework for structured assessment of fallopian tube epithelial abnormalities [8]. However, its diagnostic performance has not previously been evaluated in a substantially expanded prospective cohort.

The present study therefore aimed to explore the diagnostic performance of ex vivo fallopian tube cytology and the CytoSaLPs score in women undergoing salpingectomy or salpingo-oophorectomy. Specifically, we aimed to correlate cytological findings with histopathology, evaluate specimen-level diagnostic performance, and explore the discriminatory ability of the CytoSaLPs score using receiver operating characteristic analysis. Because the score was developed and evaluated within the same study cohort, the present investigation should be considered an exploratory proof-of-concept study rather than a formal validation study.

## 2. Methods

### 2.1. Study Design and Setting

This was a prospective single-center observational study conducted at the Gynecologic Oncology Department and Cytopathology and Pathology Laboratories of Metaxa Memorial Cancer Hospital of Piraeus, Greece, between January 2020 and December 2023. Because the study was conducted at a tertiary gynecologic oncology referral center, the prevalence of malignant disease was substantially higher than would be expected in the general population. Consequently, predictive values derived from this cohort should not be extrapolated directly to average-risk screening populations.

### 2.2. Study Population

Women undergoing salpingectomy or salpingo-oophorectomy for benign, premalignant, or malignant gynecologic indications were eligible for inclusion. Exclusion criteria included severely damaged fallopian tubes precluding adequate brushing, prior formalin fixation before cytological sampling, inadequate cellular preservation, mechanically disrupted fimbrial anatomy, and inability to obtain informed consent. A total of 304 women were enrolled.

### 2.3. Ethical Approval and Patient Consent

The study was conducted in accordance with the Declaration of Helsinki and was approved by the Bioethics Committee of the National and Kapodistrian University of Athens on 9 March 2021 with project identification code 302/09-03-2021 and by the Scientific Council of Metaxas General Hospital on 8 July 2020 with project identification code 10612-9/7/2020.

Informed consent was obtained from all subjects involved in the study.

### 2.4. Specimen Flow and Unit of Analysis

Because bilateral adnexal surgery was performed in many participants, the number of surgical specimens exceeded the number of enrolled patients.

The primary unit of analysis for cytological diagnostic performance calculations was the individual fallopian tube specimen. Fallopian tube cytological findings were compared separately with the corresponding histopathological findings of the fallopian tube and ovary. No ovarian cytological specimens were collected. The ovarian analyses therefore represent comparisons between fallopian tube cytology and ovarian histology, rather than ovarian cytology. The purpose was to assess whether cytological abnormalities obtained from the fallopian tube were associated with histological malignancy in the corresponding ovary.

A total of 544 paired specimens were initially available for cytology–histology correlation. Of these, 53 specimens were classified as non-diagnostic, primarily because of insufficient cellularity or obscuring material. The primary diagnostic performance analysis therefore included 491 evaluable paired specimens. Thus, the study population progressed as follows:

304 patients → 544 paired specimens available for cytology–histology correlation → 53 non-diagnostic specimens → 491 specimens included in the primary diagnostic performance analysis.

Because this was an exploratory proof-of-concept study primarily evaluating specimen-level cytomorphology and because bilateral specimens obtained from the same patient are not statistically independent, specimen-level analyses may underestimate the uncertainty around diagnostic performance estimates. Patient-level bootstrap procedures were therefore used to account for within-patient clustering in estimation of confidence intervals.

### 2.5. Fallopian Tube Cell Collection Procedure

**Figure 1:**
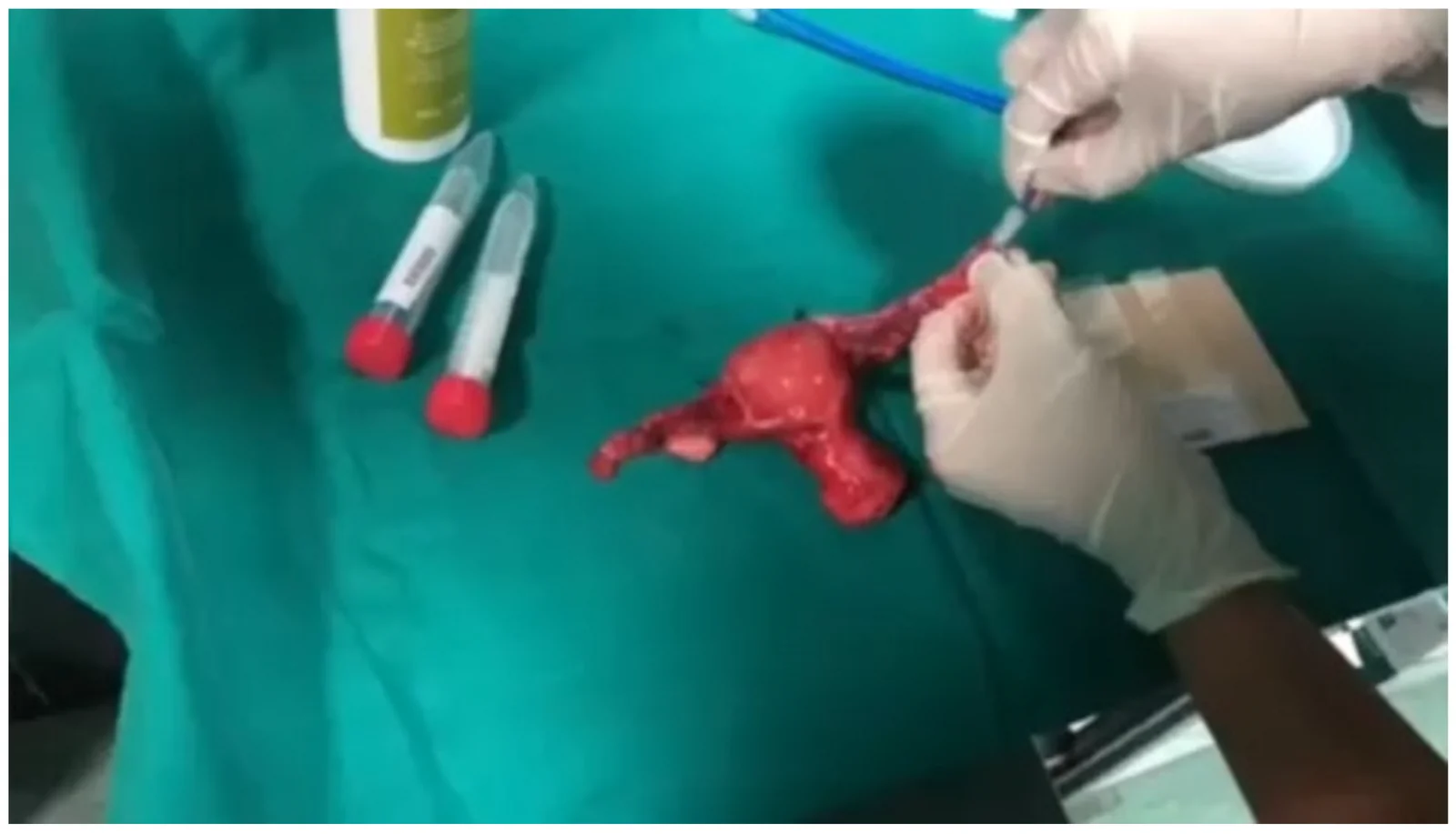
Insertion of the brush with the help of small forceps into the fallopian tube lumen through the tip of the cilia.

**Figure 2:**
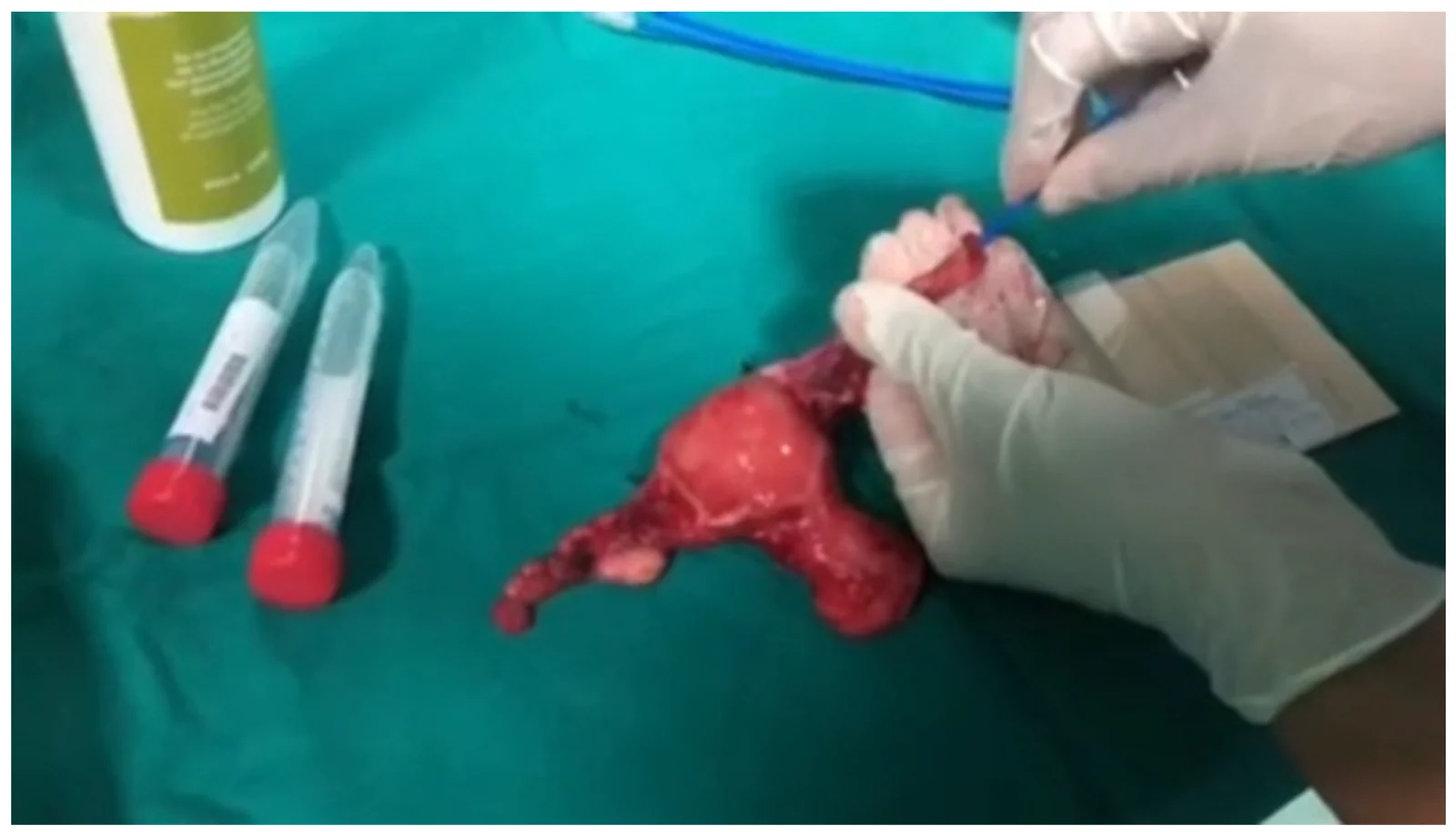
Move the brush until it reaches the isthmus. Then, slowly rotate and extract the brush.

**Figure 3:**
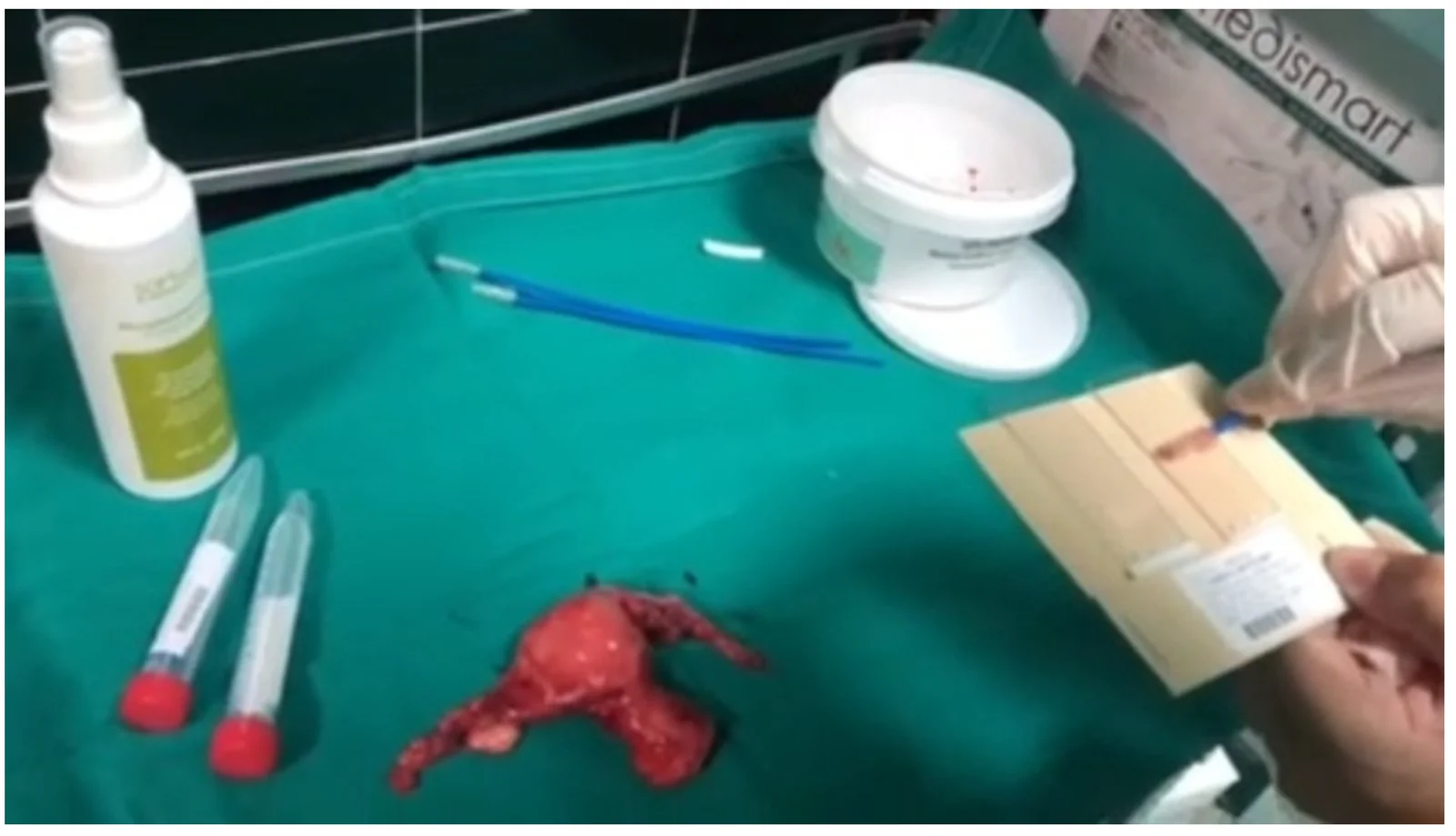
Coating on a slide.

**Figure 4:**
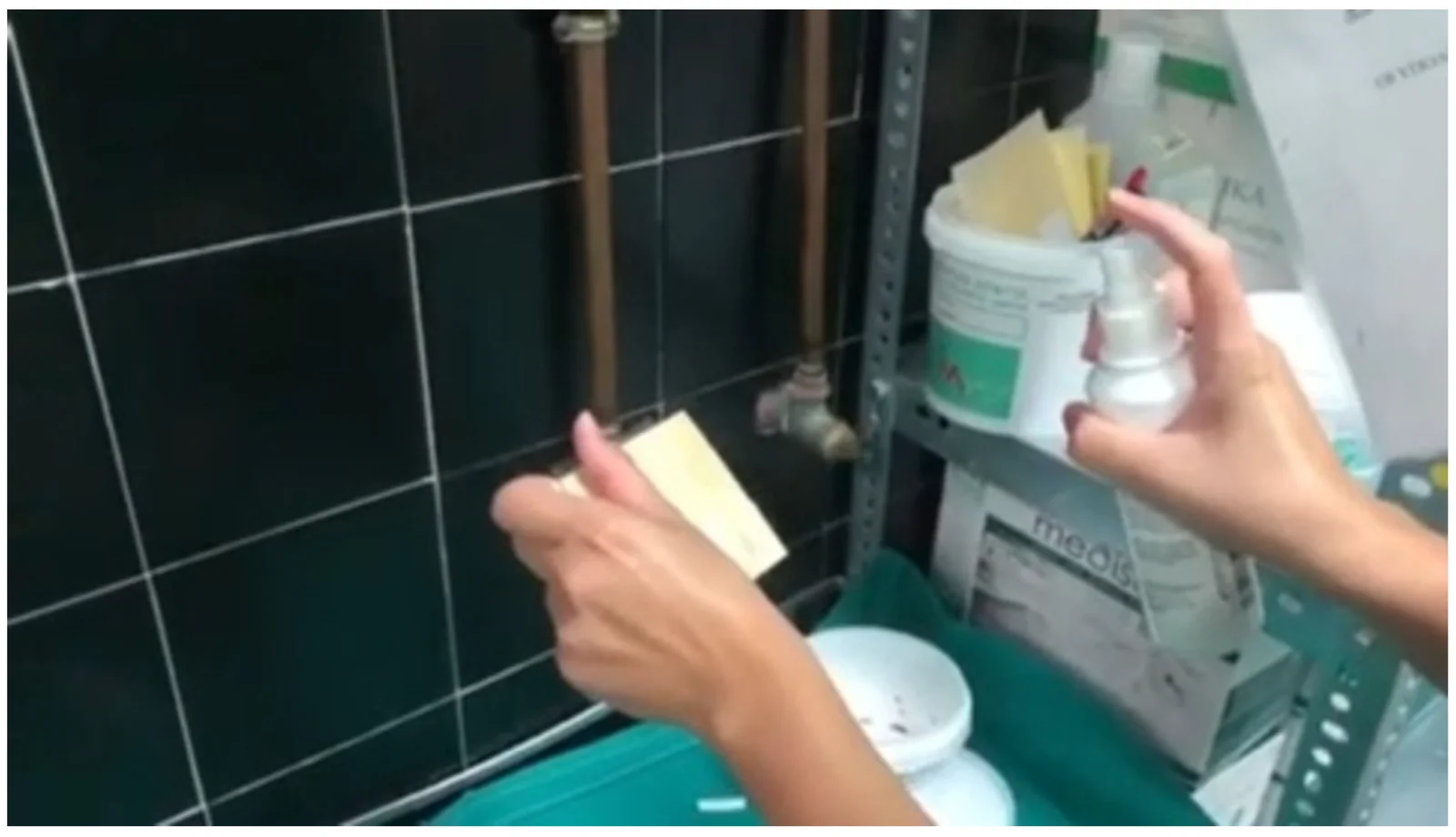
Spraying with fixing material.

**Figure 5:**
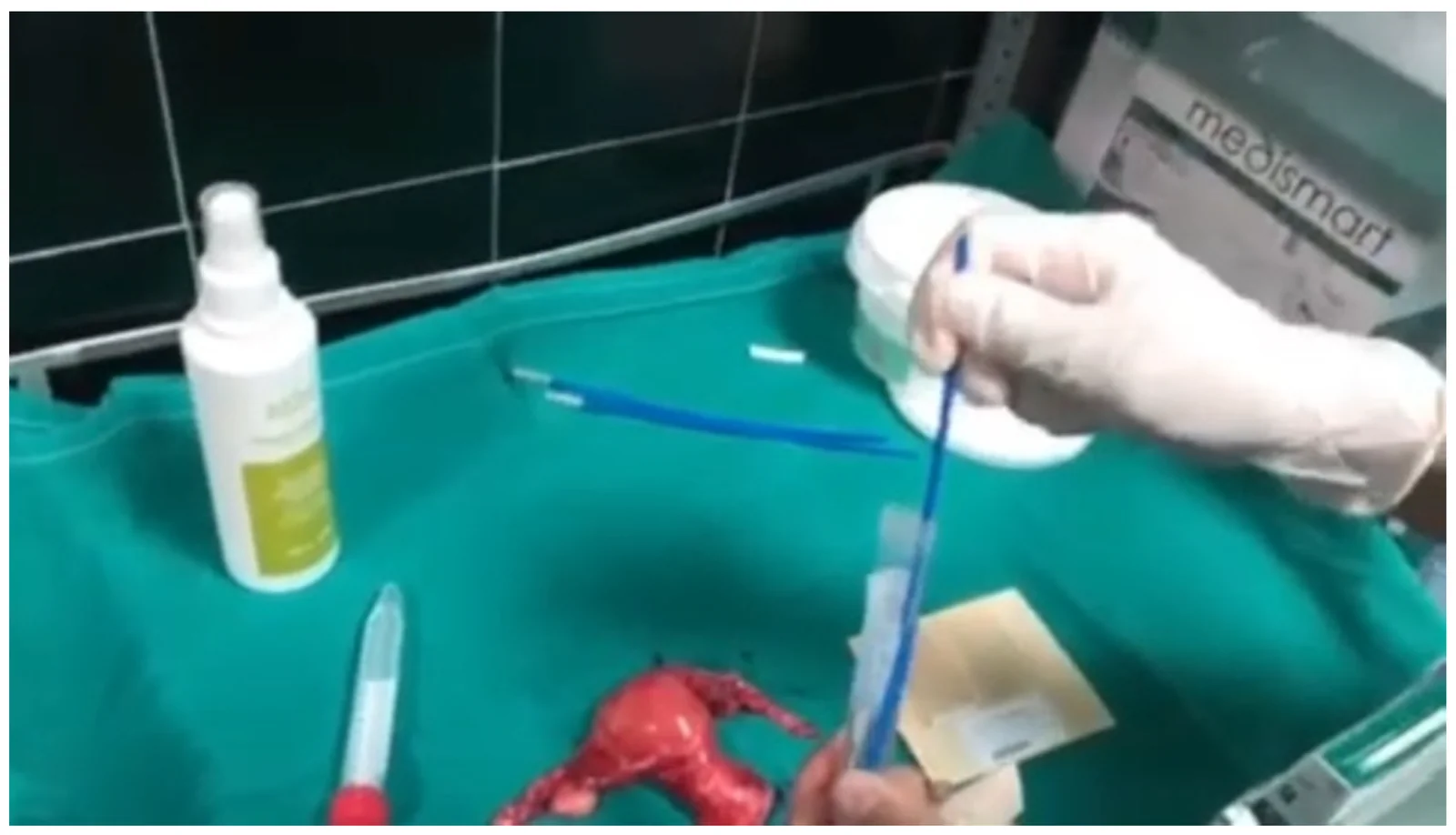
Rinsing the brush in the Cytolyt preservative solution.

Cytological samples were collected ex vivo within 30 min of surgical removal and before formalin fixation. A sterile disposable cytobrush with a diameter of approximately 2 mm was introduced through the fimbrial end of the fallopian tube and advanced toward the ampullary-isthmic region. Two to three rotational passes were performed while the brush was slowly withdrawn to maximize epithelial cell collection while minimizing mechanical trauma. Sampling primarily targeted the fimbrial and distal ampullary epithelium because of the recognized role of the distal fallopian tube in serous carcinogenesis. Bilateral tubes were sampled separately using independent brushes and separately labeled collection containers. Conventional smears were immediately fixed using cytological spray fixative. Residual cellular material was subsequently transferred into preservative solution for liquid-based cytological preparation and the samples were stored at 4 °C until processing. Slides were prepared using an automated filter-based processor stained according to the standard Papanicolaou technique (Technique I) using hematoxylin, Orange G, and EA counterstains. Sample were considered adequate when identifiable epithelial cellular groups with sufficiently preserved morphology were present and severe obscuring blood or degeneration was absent (Figure 1, Figure 2, Figure 3, Figure 4 and Figure 5).

### 2.6. Histopathological Examination

Histological examination was used as the reference standard for diagnostic performance analyses. Fallopian tube specimens were processed according to the SEE-FIM protocol [9], enabling detailed examination of the fimbrial epithelium and identification of occult lesions, including STIC. For diagnostic analyses, histologically confirmed borderline tumors and invasive malignancies were considered positive findings. STIC lesions were additionally described separately because of their biological relevance as precursor lesions.

### 2.7. Cytological Evaluation

**Figure 6:**
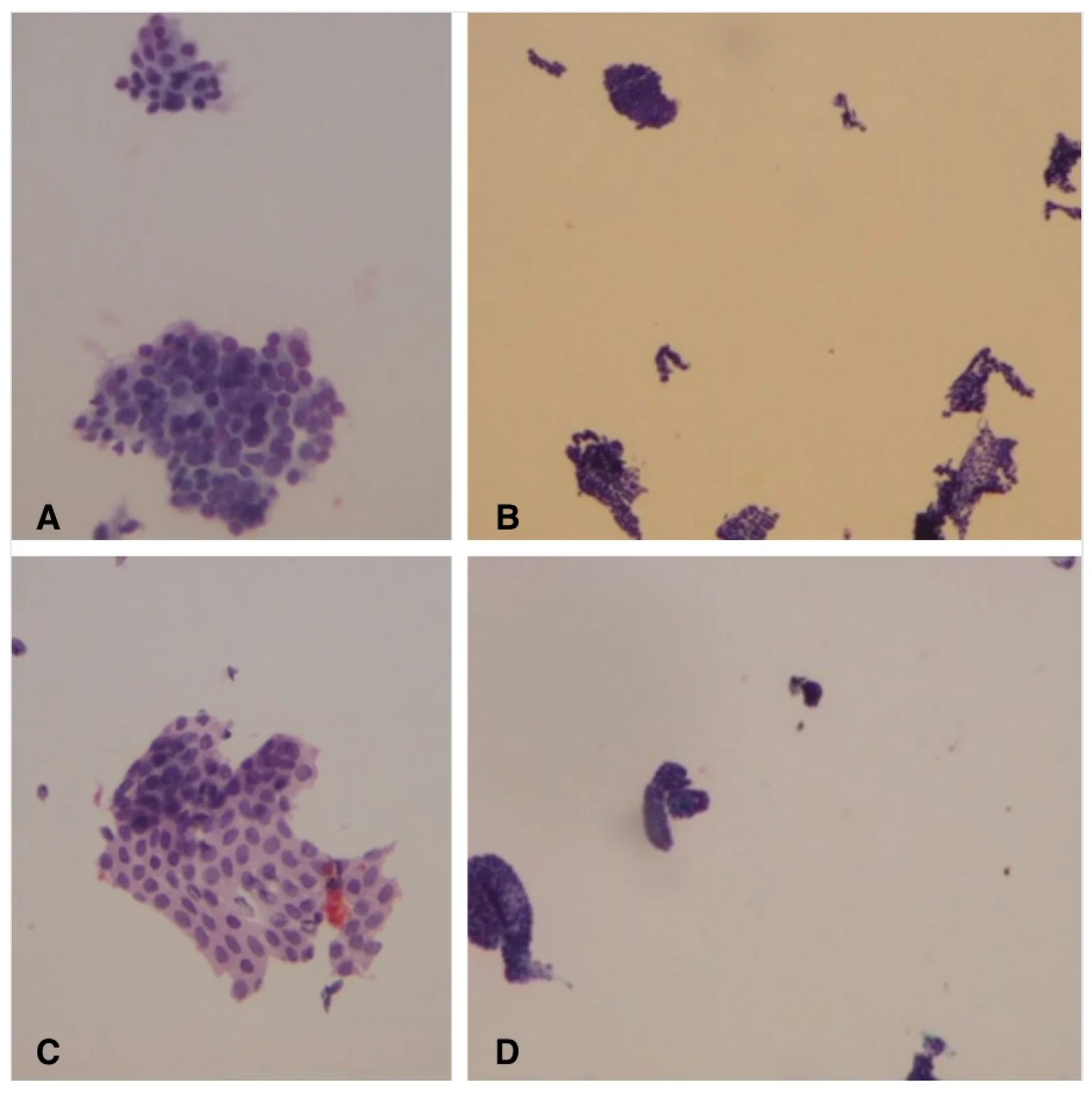
Cytological findings in thin prep preparations of fallopian tube cells (**A**). Thin prep × 20. Groups of mesothelial cells. (**B**) Thin prep × 10. Single cells of normal fallopian tube. (**C**) Thin prep × 10. Three-dimensional cell clusters with papillary configuration in serous carcinoma. (**D**) Thin prep × 20. Branching groups of cells with disruption of cellular architecture in atypia.

The cytological morphology of fallopian tube epithelial cells was assessed and cases were classified into the following diagnostic categories: *benign*, *atypical*, *severe atypia*, *suspicious for malignancy*, *malignant* or *non-diagnostic*. The primary unit of analysis for cytological diagnostic performance calculations was the individual fallopian tube specimen. Because bilateral specimens were obtained from many patients, left and right specimens were initially analyzed separately and subsequently combined for the relevant analyses. Cases classified as “atypia” demonstrated cytological abnormalities exceeding those expected from purely reactive changes but insufficient for definitive classification as malignant. All cytological slides were independently reviewed by two experienced cytopathologists who were blinded to the corresponding histological findings. In cases of disagreement, a consensus diagnosis was reached through joint review. Some of the cytological features evaluated are included in Figure 6. Non-diagnostic samples primarily reflected insufficient cellularity or obscuring blood and were excluded from the primary sensitivity and specificity calculations. Their frequency and histological outcomes were reported separately. Formal interobserver agreement statistics, such as Cohen’s kappa, were not calculated.

#### Definition of Cytological Positivity

For the primary binary diagnostic analysis, cytological findings were dichotomized into negative and positive categories. Benign cytology was classified as negative, whereas atypia, severe atypia, suspicious for malignancy, and malignant cytology were classified as positive. Non-diagnostic specimens were excluded from the primary diagnostic performance analysis.

### 2.8. Cytosalps Score Development

**Figure 7:**
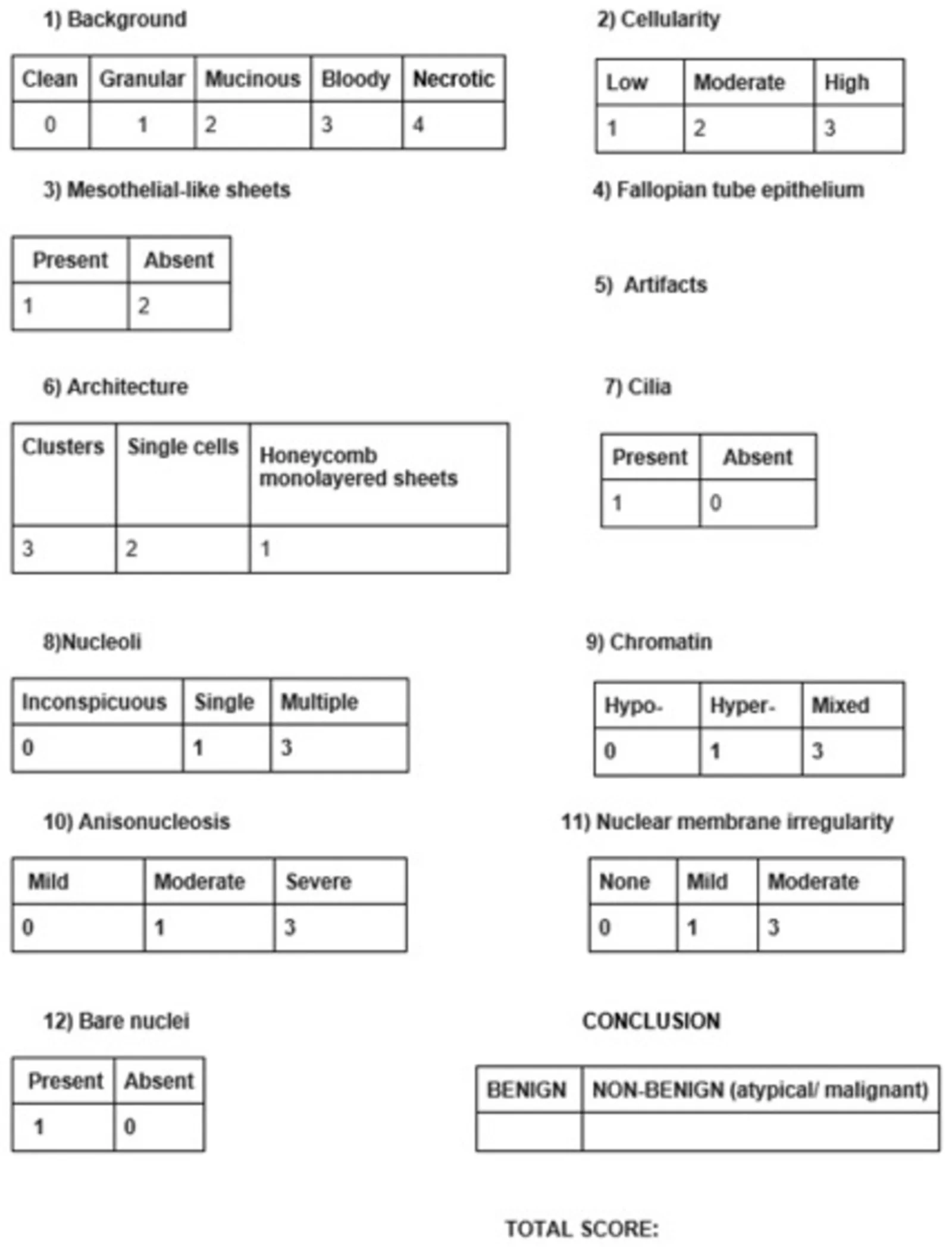
The CytoSaLPs score.

The CytoSaLPs score was developed as a structured cytomorphological framework to quantify cellular features considered relevant to the interpretation of fallopian tube cytology. Score development was based on classical cytological principles, the published literature, expert consensus and empirical weighting [10,11,12,13,14,15,16,17,18,19]. Individual cytological features were assigned numerical values, generating a total possible score from 3 to 29 (Figure 7). Ex vivo sampling was intentionally used during this developmental phase to standardize specimen acquisition and permit direct correlation between cytology and histopathology before future investigation of clinically applicable in vivo sampling methods. Because the score was developed and evaluated within the same cohort, its performance estimates and ROC-derived thresholds should be considered exploratory and provisional rather than validated.

### 2.9. Statistical Analysis

Data were recorded in Microsoft Excel using SAS version 9.4 for Windows (SAS Institute Inc., Cary, NC, USA) and R language version 4.5.1 (R Foundation for Statistical Computing, Vienna, Austria). Receiver operating characteristic curves were constructed to evaluate the discriminatory ability of the CytoSaLPs score. The area under the ROC curve (AUC) and corresponding 95% confidence intervals were calculated. Optimal thresholds were identified using the maximum Youden Index. Because the same cohort was used to develop the score and derive its optimal thresholds, no claim of independent validation is made. To account for the clustering of multiple observations within the same patient, confidence intervals were estimated using a patient-level bootstrap procedure. Patients were resampled with replacement, while all observations belonging to the same patient were retained within each bootstrap sample. Diagnostic performance measures, including sensitivity, specificity, positive predictive value, negative predictive value and the other performance metrics, were recalculated for each bootstrap sample. The AUC was estimated from the original sample, whereas its standard error was estimated using patient-level bootstrap resampling. The 95% confidence interval was obtained using the percentile bootstrap method, and the *p*-value for AUC = 0.50 was calculated using a Wald-type test based on the bootstrap-estimated standard error. A total of 2000 bootstrap replicates were used.

## 3. Results

### 3.1. Study Population and Specimen Flow

A total of 304 women were included in the study. Because bilateral fallopian tubes and ovaries were sampled whenever available, the number of specimens exceeded the number of patients. Overall, 544 paired cytology–histology specimens were available for initial assessment. Fifty-three specimens were classified as non-diagnostic. Consequently, 491 paired specimens were included in the primary diagnostic performance analysis. Patient demographics, clinical characteristics, surgical indications, procedures, cytological findings, and histopathological findings are summarized in Table 1.

### 3.2. Non-Diagnostic Cytological Samples

A total of 54 cytological samples were classified as non-diagnostic, because of insufficient cellularity or obscuring blood and/or degenerative changes. Of these, 53 specimens had a corresponding histological reference standard and were therefore included in the paired cytology–histology analysis as non-diagnostic cytological samples. Among the 53 non-diagnostic specimens with available histology, one (1.9%) was associated with malignancy. One non-diagnostic cytological specimen did not have a corresponding histological specimen and was therefore not included in the diagnostic accuracy analysis. Thus, the discrepancy between the total number of non-diagnostic cytological specimens (*n* = 54) and those included in the paired analysis (*n* = 53) reflects the absence of a corresponding histological reference standard in one case.

Non-diagnostic specimens were excluded from the primary calculations of sensitivity, specificity, positive predictive value, negative predictive value, and overall accuracy because a definitive cytological classification could not be assigned. However, their frequency was reported separately because the occurrence of non-diagnostic samples is clinically relevant to the potential future application of this sampling approach. The exclusion of non-diagnostic specimens from the primary diagnostic accuracy analysis may result in estimates that are more favorable than those observed in routine clinical or screening settings and is therefore considered an important limitation of the present study.

### 3.3. Correlation Between Cytology and Histology

Cytological findings of the fallopian tubes were compared with the corresponding histological diagnoses of the fallopian tube and ovary.

For fallopian tube specimens, the proportion of histologically malignant cases increased across more abnormal cytological categories. Benign cytology was rarely associated with histologically confirmed malignancy, whereas atypical, suspicious, and malignant cytological findings showed progressively higher proportions of malignant histology.

A similar pattern was observed when fallopian tube cytology was compared with ovarian histology, although the association between cytological abnormalities and histologically confirmed ovarian malignancy was less pronounced than for the corresponding fallopian tube histology.

The detailed cross-tabulations are presented in Table 2.

### 3.4. Serous Tubal Intraepithelial Carcinoma

Three cases of STIC were identified histologically using the SEE-FIM protocol. All lesions were located within macroscopically normal fimbrial epithelium. Two of the three cases demonstrated cytological abnormalities and elevated CytoSaLPs scores above the provisional diagnostic threshold, with scores of 17 and 19. One specimen was classified as non-diagnostic because of insufficient cellularity. Because of the small number of STIC lesions, no formal conclusions regarding the sensitivity of fallopian tube cytology for detecting STIC can be drawn. These findings should therefore be interpreted as descriptive and hypothesis-generating.

### 3.5. Diagnostic Performance of Cytology

Diagnostic performance was assessed using histopathology as the reference standard. After exclusion of non-diagnostic specimens, 491 paired specimens were included in the primary analysis. Cytological positivity was defined as atypia, severe atypia, suspicious for malignancy, or malignant cytology, whereas benign cytology was considered negative. Each anatomical site was evaluated on a specimen basis. For fallopian tube cytology, there were 34 true-positive, 323 true-negative, 132 false-positive, and two false-negative results. The resulting sensitivity was 94.4%, specificity was 71.0%, and overall accuracy was 72.7%. When fallopian tube cytology was evaluated against ovarian histology, the diagnostic performance was lower than that observed for the correlation with fallopian tube histology, with 51 true-positive, 305 true-negative, 116 false-positive, and 19 false-negative results. Sensitivity was 72.9%, specificity was 72.4%, and overall accuracy was 72.5%. For combined adnexal analysis, there were 52 true-positive, 157 true-negative, 65 false-positive, and 16 false-negative results. Sensitivity was 76.5%, specificity was 70.7%, and overall accuracy was 72.1%. The TP, TN, FP, and FN values are presented in Table 3. The diagnostic performance of fallopian tube cytology is shown in Table 4. The performance of cytology when cytological atypias are considered correct when they are histologically confirmed as negative is presented in the Supplementary Materials.

### 3.6. CytoSaLPs Score and ROC Analysis

Receiver operating characteristic analysis was performed to explore the discriminatory ability of the CytoSaLPs score. For fallopian tube malignancy, the score demonstrated good discrimination. The AUC was 0.8072 (95% CI: 0.6702–0.9442, DeLong method) for the left fallopian tube and 0.8072 (95% CI: 0.6702–0.9442, DeLong method) for the right fallopian tube. There was no statistically significant difference between the two sides (*p* = 0.3086). When both sides were combined, the AUC was 0.8534 (95%CI 0.7581–0.930), after adjustment for observations from the same patient, indicating good discriminatory ability (*p* < 0.0001). Using the maximum Youden Index, the optimal threshold in this cohort was a score of 16.5, corresponding to a sensitivity of 72.2% and specificity of 85.6%. Because this threshold was derived from the same cohort in which the score was developed and evaluated, it should be regarded as provisional rather than validated.

When the fallopian tube CytoSaLPs score was evaluated against ovarian histology, moderate discriminatory performance was observed. The aggregated analysis demonstrated an AUC of 0.6794 (95% CI 0.5869–0.7688; *p* = 0.0001, adjusted for observations from the same patients). The exploratory optimal threshold was 15.5, corresponding to a sensitivity of 55.1% and specificity of 78.6%.

**Figure 8:**
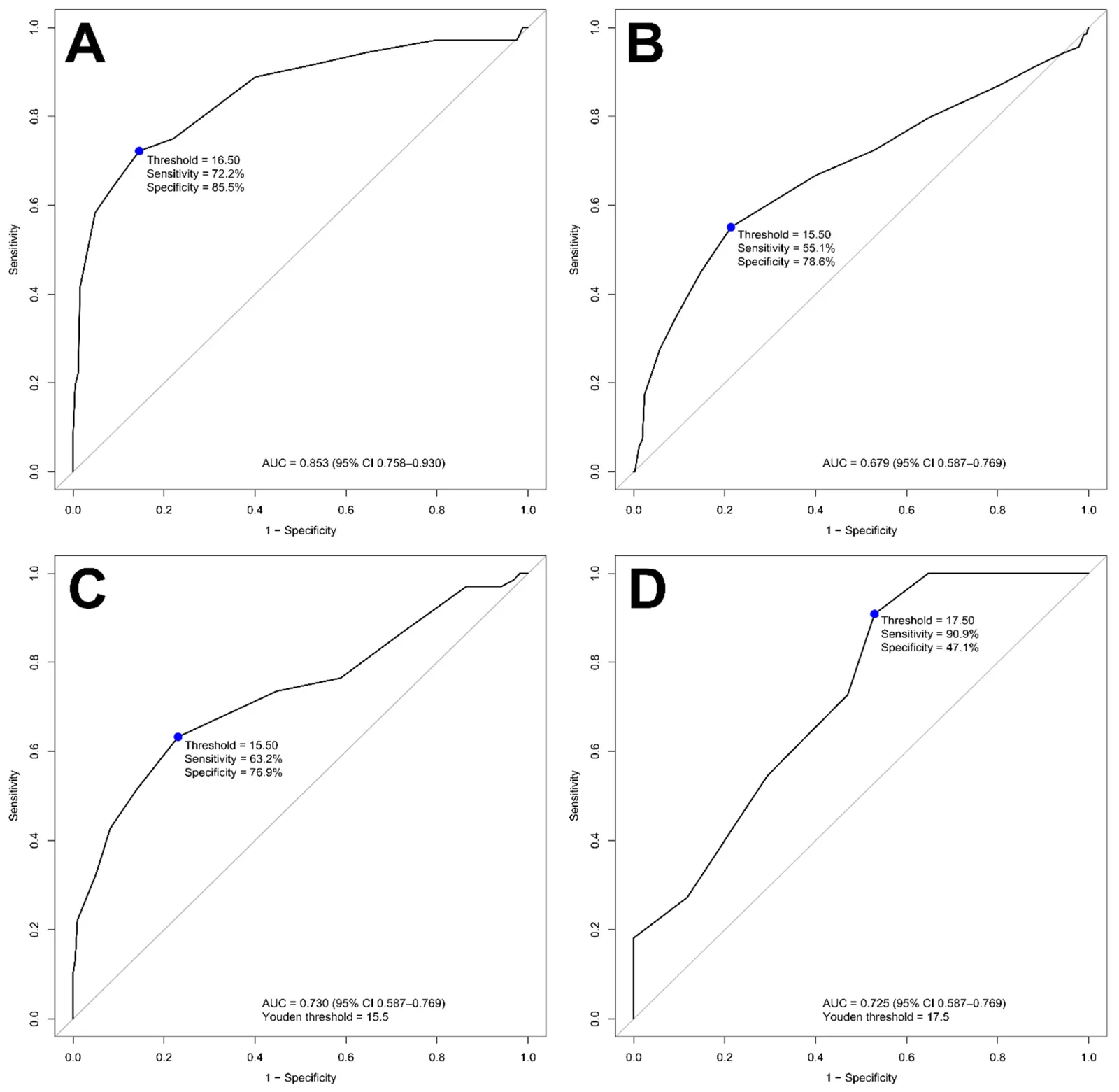
ROC curves for cytological scores and histological outcomes. (**A**) ROC curve for the cytological score and the histological outcome of the fallopian tube. The blue points indicate the threshold (optimal score) where sensitivity is as close as possible to specificity. (**B**) ROC curve for the cytological score and the histological outcome of the ovary. Histological score appears to be related to its histological diagnosis. (**C**) ROC curve for the cytological score and the histological outcome of the adnexa. The area under the curve (AUC) is 73.0% (95% CI: 58.7—76.9%, *p* < 0.0001). For a threshold of 15.5, sensitivity is 63.2% and specificity is 76.9%. (**D**) ROC curve for the cytological score and the histological outcome of the peritoneum. Despite only 28 results, the histological score appears to have potential to its histological diagnosis with AUC = 72.5% (95% CI: 58.7—76.9%, *p* = 0.019).

For the combined adnexal analysis, the final patient score was derived from the highest score obtained from bilateral specimens. Given that histological results were also available for the peritoneum, the correlation of the cytological score with the histological diagnosis of the peritoneum was examined. Receiver operating characteristic curves for all anatomical sites are presented in Figure 8.

## 4. Discussion

### 4.1. Principal Findings

In this prospective exploratory study, ex vivo fallopian tube cytology demonstrated high sensitivity for histologically confirmed tubal malignancy, whereas specificity was moderate. The CytoSaLPs score provided a structured framework for evaluating cytomorphological abnormalities and demonstrated good discriminatory ability for fallopian tube malignancy, with a combined AUC of 0.8534 (95% CI: 0.7581–0.9300) after adjustment for observations from the same patient.

Diagnostic performance was more modest when fallopian tube cytology was compared with ovarian histology and in the combined adnexal analysis. This finding is biologically plausible because not all ovarian malignancies arise from the fallopian tube epithelium, and tubal cytological abnormalities may therefore be less pronounced in non-serous or true ovarian tumors.

Three STIC lesions were identified using the SEE-FIM protocol. Two demonstrated cytological abnormalities and elevated CytoSaLPs scores, whereas one specimen was non-diagnostic. Although these findings are consistent with the biological rationale for examining the distal fallopian tube, the number of lesions was insufficient to evaluate diagnostic performance for STIC.

### 4.2. Comparison with Previous Studies

Lee et al. highlighted the increasing recognition of STIC as a precursor to high-grade serous carcinoma and discussed the potential implications of identifying these lesions for early detection and prevention strategies [20]. Previous studies have demonstrated the feasibility of obtaining cytological specimens from the fallopian tube and have identified morphological characteristics associated with malignant disease. Chen et al. evaluated ex vivo fallopian tube brush specimens and reported associations between cytological features and high-grade serous carcinoma and/or STIC [21]. Dhanani et al. characterized the cytomorphological appearance of benign fimbrial epithelium and highlighted features that may assist in distinguishing benign tubal cells from potential malignant lesions [22]. However, previous investigations have generally been limited by relatively small sample sizes and the absence of standardized reporting systems. The present study extends these observations by evaluating a larger prospective surgical cohort and introducing a structured cytological scoring framework. Direct comparisons between studies remain challenging because of differences in sampling methods, patient populations, diagnostic thresholds, and definitions of cytological abnormality.

### 4.3. Interpretation of Diagnostic Performance

The high sensitivity observed for fallopian tube cytology should be interpreted cautiously. First, the study population was recruited from a tertiary gynecologic oncology referral center and therefore included a substantially higher prevalence of malignant disease than would be expected in an average-risk screening population. Second, the primary diagnostic analysis excluded non-diagnostic specimens. Consequently, the reported sensitivity and negative predictive value represent performance among evaluable samples rather than the performance of the entire sampling procedure. In the present cohort, approximately 10% of specimens were non-diagnostic. This proportion is clinically relevant because any future screening or minimally invasive diagnostic approach would need to account for failed or inadequate sampling. Third, bilateral specimens were obtained from some patients. Although patient-level bootstrap procedures were used to account for within-patient clustering when estimating uncertainty, the primary diagnostic analyses remained specimen-based. Therefore, the apparently high sensitivity and negative predictive value should not be interpreted as evidence that fallopian tube cytology is currently suitable as a screening test.

### 4.4. CytoSaLPs Score

The CytoSaLPs score demonstrated good discriminatory ability for fallopian tube malignancy, with a combined AUC of 0.8534 (95% CI 0.7581–0.9300) after adjustment for observations from the same patient. However, an important limitation is that the score was developed and evaluated within the same cohort. The ROC-derived thresholds were also selected using the same dataset. This may lead to optimistic estimates of diagnostic performance and represents a potential source of overfitting. Accordingly, the present study does not constitute external validation of the CytoSaLPs score. The score and its proposed thresholds should be considered exploratory and provisional. Future studies should evaluate the score in independent multicenter cohorts and assess both interobserver reproducibility and calibration. Internal validation using bootstrap resampling or cross-validation may provide an intermediate assessment of robustness if the underlying dataset is available.

### 4.5. Strengths and Limitations

The main strengths of this study include its prospective design, the relatively large number of cytological specimens, standardized ex vivo sampling, blinded cytological evaluation and direct correlation with detailed histopathological examination. The use of the SEE-FIM protocol also allowed detailed examination of the distal fallopian tube and identification of occult STIC lesions.

Several limitations should be acknowledged. First, this study was performed at a tertiary gynecologic oncology center, with an enriched prevalence of malignancy, limiting generalizability to screening populations. Second, the CytoSaLPs score was developed and evaluated within the same cohort, and the diagnostic thresholds were selected using the same ROC analysis. Third, bilateral specimens were included in the specimen-level analysis. Although patient-level bootstrap resampling was used to account for within-patient clustering in the estimation of confidence intervals, the study was not designed as a fully patient-level diagnostic study. Fourth, approximately 10% of samples were non-diagnostic and were excluded from the primary diagnostic calculations. Although this approach allowed evaluation of the diagnostic performance of interpretable cytological samples, it may overestimate sensitivity, specificity, and negative predictive value compared with a real-world screening setting in which unsuccessful or insufficient sampling would represent an important component of overall test performance. Fifth, interobserver agreement was not formally assessed. Finally, only three STIC lesions were identified, precluding meaningful evaluation of diagnostic sensitivity for precursor lesions. Additional methodological challenges should also be considered. Interpretation of tubal cytology remains difficult because cellular atypia may closely resemble adenocarcinoma and may reflect either reactive or neoplastic changes, while mixed epithelial, mesothelial, inflammatory, and degenerative cell populations may reduce diagnostic reproducibility. Currently, atypical tubal findings have no established clinical implications or recommended management strategies [23,24]. Furthermore, advanced pelvic carcinomatosis may obscure the true site of origin despite comprehensive SEE-FIM examination. Consequently, some tumors classified histologically as primary ovarian carcinomas may have originated within the distal fallopian tube, potentially influencing the calculated diagnostic performance of tubal cytology.

### 4.6. Clinical and Research Implications

The present findings should not be interpreted as supporting routine clinical screening using fallopian tube cytology. The CytoSaLPs score demonstrated higher values in high-grade serous carcinomas compared with non-serous subtypes, consistent with the dualistic model of ovarian carcinogenesis and the proposed tubal origin of type II serous carcinomas (Table 5).

High-grade serous carcinomas frequently exhibited cytomorphologic features contributing to higher scores. In contrast, mucinous and endometrioid tumors often demonstrated less pronounced cytological abnormalities within the tubal epithelium, potentially limiting the diagnostic sensitivity of tubal cytology for these histotypes. Because the study was not powered for subtype-specific diagnostic analyses, these observations should be considered exploratory. Instead, the study provides proof-of-concept evidence that cytological abnormalities obtained from the fallopian tube may correlate with histologically confirmed malignancy and that a structured scoring system may facilitate standardized assessment. Future research should focus on independent external validation of the CytoSaLPs score, assessment of interobserver reproducibility, clustered or patient-level analyses accounting for bilateral specimens, evaluation of clinically applicable in vivo sampling methods, prospective assessment in populations with different baseline risks and integration of cytology with molecular biomarkers. Ongoing prospective studies, such as the tubal lavage study by Gizzo et al. [25], are investigating cytological evaluation in women with and without BRCA1/2 mutations, correlating cytology with serum biomarkers, imaging, and histology. TP53 mutations have been shown to be detectable cytologically in tubal, endometrial, and cervical samples [26,27]. Combining cytology and molecular information may improve diagnostic performance beyond cytology alone. Potential biomarkers include TP53 mutations, BRCA-associated alterations, circulating tumor DNA, and other liquid biopsy approaches.

Recent advances in hysteroscopic catheter-based tubal lavage suggest that outpatient sampling of tubal epithelial cells may eventually become feasible [27]. Minimally invasive approaches, including hysteroscopic or catheter-based tubal sampling, may eventually permit prospective in vivo evaluation of tubal epithelial cells, particularly in women in whom salpingectomy is not otherwise required, such as during cesarean sections, tubal ligations, endometriosis surgery, diagnostic laparoscopies, appendectomies, and other pelvic procedures, including minimally invasive outpatient interventions such as hysteroscopy [28,29,30]. However, the feasibility, safety, reproducibility, and clinical utility of such approaches remain to be established.

## 5. Conclusions

This prospective exploratory study demonstrates that ex vivo fallopian tube cytology can detect cytological abnormalities associated with histologically confirmed tubal malignancy and that the CytoSaLPs score provides a structured framework for evaluating these abnormalities. Fallopian tube cytology showed high sensitivity in the evaluable specimen population, whereas specificity was moderate. The CytoSaLPs score demonstrated good discriminatory ability for fallopian tube malignancy but more modest performance when evaluated against ovarian histology and in combined adnexal analyses. However, the findings should be interpreted cautiously. The study was conducted in a tertiary gynecologic oncology population under ex vivo conditions, non-diagnostic samples were excluded from the primary diagnostic performance analysis, bilateral specimens were obtained from some patients, and the CytoSaLPs score and its diagnostic thresholds were developed and evaluated within the same cohort. Therefore, the proposed score and cut-offs should be considered exploratory and provisional. Independent multicenter validation, assessment of reproducibility, appropriate patient-level or clustered analyses, and development of clinically applicable in vivo sampling methods will be required before any clinical application can be considered.

## Figures and Tables

**Table 1:** Patient demographics, clinical characteristics, surgical indications, procedures, and histopathological findings (N = 304). Specimen counts exceed patient counts because bilateral specimens were analyzed separately (percentages and other descriptive statistics are calculated after excluding missing data).

Category	Subcategory/Organ	*n* (%) or Mean ± SD	Median [Min–Max]	Missing *n* (%)
Age (years)	—	56.7 ± 13.3	56 [12–88]	5 (1.6)
Marital Status	Single	22 (8.3%)	—	40 (13.2)
	Divorced	20 ((7.6%)	—	
	Married	198 (75.0%)	—	
	Widowed	24 (9.1%)	—	
Nationality	Greek	237 (78.0)	—	—
	Albanian	14 (4.6)	—	
	Other	53 (17.4)	—	
Menarche (years)	—	12.6 ± 1.46	13 [8–17]	37 (12.2)
Menopause (years)	—	49.3 ± 6.16	50 [0–58]	140 (46.1)
Cycle disorders	Yes	61 (22.8%)	—	37 (12.2)
	No	206 (77.2%)	—	
Contraceptive use	Yes	21 (7.9%)	—	37 (12.2)
	No	246 (92.1%)	—	
Hormone therapy	Yes	24 (9.0%)	—	37 (12.2)
	No	243 (91.0%)	—	
IVF	Yes	10 (3.8%)	—	38 (12.5)
	No	256 (96.2%)	—	
History of Cancer	Breast	20 (7.5)	—	—
	Ovarian/Fallopian	9 (3.4%)	—	—
	Other BRCA-related	11 (4.1%)	—	—
Appendectomy	Yes	77 (28.7%)	—	—
Surgical Indication	Pelvic mass	211 (69.4)	—	—
	Endometrial cancer	39 (12.8)	—	—
	Ovarian cancer	19 (6.3)	—	—
	Fallopian tube cancer	7 (2.3)	—	—
	Cervical cancer	5 (1.6)	—	—
	Uterine sarcoma	5 (1.6)	—	—
	Other	18 (5.9)	—	—
Type of Surgery	TAH + BSO	187 (61.5)	—	—
	PDS	48 (15.8)	—	—
	IDS	12 (3.9)	—	—
	Adnexectomy/Bilateral	23 (7.5)	—	—
	TAH + BSO ± omentectomy/LND/appendectomy	29 (9.5)	—	—
	Radical hysterectomy ± LND/omentectomy	5 (1.6)	—	—
Fallopian Tube Cytology (both sides *n* = 608)	Benign	330 (59.9%)	—	57 (9.4%)
	Atypia	124 (22.5%)		
	Severe atypia	7 (1.3%)		
	Suspect for malignancy	2 (0.4%)	—	—
	Malignancy	34 (6.2%)	—	—
	Non-diagnostic	54 (9.8%)	—	—
Fallopian Tube Cytology Score (both sides *n* = 608)	—	14.0 ± 3.0	14 [6–24]	113 (18.6%)
Fallopian Tube Histology (both sides *n* = 608)	Benign	532 (92.0%)	—	30 (4.9)
	Malignancy	42 (7.3%)	—	
	Borderline/STIC	4 (0.7%)	—	—
Ovary Histology (both sides *n* = 608)	Benign	488 (84.6%)	—	31 (5.1)
	Malignancy	78 (13.5%)	—	—
	Borderline	11 (1.9%)	—	—
Adnexa (Tube + Ovary) (*n* = 304)	Benign	231 (76.0)	—	—
	Malignancy	73 (24.0)	—	—
Peritoneum Histology (*n* = 304)	Benign	19 (63.3%)	—	274 (90.1)
	Malignant	45 (14.8)	—	—
	Precancerous	5 (1.6)	—	—
Endometrium Histology (*n* = 304)	Benign	194 (79.5%)	—	60 (19.7)
	Malignancy	45 (18.4%)	—	—
	Precancerous	5 (2.0%)	—	—

**Table 2:** Crosstabulation of fallopian tube cytology and reference histology for fallopian tubes, ovaries and adnexa (left, right, and combined).

Organ/Side	Fallopian Tube Cytology	Histology: Benign	Histology: Borderline Malignancy	Histology: Malignant	Histology: Malignant/STIC	Grand Total	Proportion of Cases with Malignancy (%)
Left Fallopian Tube	Non-diagnostic	24	0	2	1	27	11.1
	Benign	165	0	2	0	167	1.2
	Atypia	55	1	4	1	61	9.8
	Severe Atypia	3	0	0	0	3	0.0
Right Fallopian Tube	Non-diagnostic	25	—	1	—	26	3.8
	Benign	158	—	0	—	158	0.0
	Atypia	56	—	6	—	62	9.7
	Severe Atypia	2	—	0	—	2	0.0
	Suspicious	3	—	1	—	4	25.0
	Malignant	7	—	13	—	20	65.0
Combined Fallopian Tube	Non-diagnostic	49	0	3	1	53	7.5
	Benign	323	0	2	0	325	0.6
	Atypia	111	1	10	1	123	8.9
	Severe Atypia	5	0	2	0	7	28.6
	Suspicious	5	0	1	0	6	16.7
	Malignant	11	0	19	0	30	63.3
Left Ovary	Non-diagnostic	24	0	3	—	27	11.1
	Benign	154	1	11	—	166	7.2
	Atypia	47	3	12	—	62	24.2
	Severe Atypia	2	0	3	—	5	60.0
	Malignant	4	0	6	—	10	60.0
	Suspected	1	1	0	—	2	50.0
Right Ovary	Non-diagnostic	22	2	2	—	26	15.4
	Benign	151	2	5	—	158	4.4
	Atypia	49	0	13	—	62	21.0
	Severe Atypia	2	0	0	—	2	0.0
	Suspicious	0	0	0	—	0	—
	Malignant	11	0	13	—	24	54.2
Combined Ovary	Non-diagnostic	46	2	5	—	53	9.4
	Benign	305	3	16	—	324	4.9
	Atypia	96	3	25	—	124	20.2
	Severe Atypia	4	0	3	—	7	42.9
	Suspicious	1	1	0	—	2	50.0
	Malignant	15	0	19	—	34	55.9
Left Adnexa	Non-diagnostic	22	—	5	—	27	18.5
	Benign	152	—	19	—	171	11.1
	Atypia	41	—	21	—	62	33.9
	Severe Atypia	2	—	3	—	5	60.0
	Suspected	0	—	2	—	2	100.0
	Malignant	1	—	9	—	10	90.0
Right Adnexa	Non-diagnostic	20	—	7	—	27	25.9
	Benign	144	—	15	—	159	9.4
	Atypia	41	—	21	—	62	33.9
	Severe Atypia	2	—	0	—	2	0.0
	Suspicious	0	—	0	—	0	—
	Malignant	1	—	23	—	24	95.8
Combined Adnexa	Non-diagnostic	42	—	12	—	54	22.2
	Benign	296	—	34	—	330	10.3
	Atypia	82	—	42	—	124	33.9%
	Severe Atypia	4	—	3	—	7	42.9%
	Suspicious	0	—	2	—	2	100.0%
	Malignant	2	—	32	—	34	94.1

**Table 3:** Diagnostic classification of fallopian tube cytology according to the histopathological reference site. True positive (TP), true negative (TN), false positive (FP), and false negative (FN) cases for the three anatomical sites of the study regarding cytological diagnosis, using the histological result as the gold standard.

Reference Site	TP	TN	FP	FN	Total
Fallopian tubes	34	323	132	2	491
Ovaries	51	305	116	19	491
Adnexa	52	157	65	16	290

TP, true positive; TN, true negative; FP, false positive; FN, false negative.

**Table 4:** Diagnostic performance of fallopian tube cytology.

Performance Measure	Fallopian Tube Cytology vs. Fallopian Tube Histology	Fallopian Tube Cytology vs. Ovarian Histology	Fallopian Tube Cytology vs. Adnexal Histology
Sensitivity	94.4% (85.7–100%)	72.9% (60.3–84.1%)	76.5% 64.6–85.9%)
Specificity	71.0% (66.2–75.8%)	72.4% (67.5–77.2%)	70.7% (64.3–76.6%)
PPV	20.5% (12.9–28.5%)	30.5% (22.4–39.1%)	44.4% (35.3–53.9%)
NPV	99.4% (98.5–100%)	94.1% (90.9–96.8%)	90.8% (85.4–94.6%)
FPR	29.0% (24.2–33.8%)	27.6% (22.8–32.5%)	29.3% (23.4–35.7%)
FNR	5.6% (0–14.3%)	27.1% (15.9–39.7%)	23.5% (14.1–35.4%)
Overall Accuracy	72.7% (68.2–77.2%)	72.5% (67.9–76.9%)	72.1% (66.5–77.2%)
PLR	3.3 (2.7–4)	2.6 (2.1–3.4)	2.6 (2–3.3)
NLR	0.1 (0–0.2)	0.4 (0.2–0.5)	0.3 (0.2–0.5)
Diagnostic Odds Ratio	41.6 (13.6–111.8)	7.1 (3.9–14.5)	7.9 (4.2–14.7)
Youden Index	65.4% (55–73.6%)	45.3% (32.1–57.2%)	47.2% (35.5–58.9%)

PPV, positive predictive value; NPV, negative predictive value; FPR, false-positive rate; FNR, false-negative rate; PLR, positive likelihood ratio; NLR, negative likelihood ratio.

**Table 5:** Histological subtypes of malignant cases and CytoSaLPs scores.

Histological Subtype	*n*	Mean CytoSaLPs Score ± SD
High-grade serous carcinoma	28	18.9 ± 3.1
Low-grade serous carcinoma	5	15.2 ± 2.8
Endometrioid carcinoma	11	14.8 ± 3.0
Clear cell carcinoma	4	13.9 ± 2.5
Mucinous carcinoma	3	12.7 ± 2.2
Metastatic/Krukenberg tumors	1	11.0
Other subtypes combined	24	14.1 ± 2.9

## Data Availability

The datasets used and/or analyzed during the current study are available from the corresponding author on reasonable request.

## Supplementary Materials

The following supporting information can be downloaded at: https://www.mdpi.com/article/10.3390/cancers18172868/s1, Table S1: True positive (TP), true negative (TN), false positive (FP), and false negative (FN) cases for the three anatomical sites of the study regarding cytological diagnosis, using the histological result as the gold standard; Table S2: Performance indicators for each anatomical site.
