# Supplementary material for: Fallopian Tube Cytology for Exploratory Detection of Adnexal Malignancy: Prospective Evaluation of the CytoSaLPs Score in an Ex Vivo Surgical Cohort
Source: Cancers (Basel). 2026 Sep 4;18(17):2868. doi: 10.3390/cancers18172868 (PMC13565569; doi:10.3390/cancers18172868)
Supplement: Supplementary file 1 [file cancers-18-02868-s001.zip › cancers-4488141-supplementary.pdf]

cancers

Supplement

Performance of cytology when cytological atypias are considered correct when they are histologically confirmed as negative.

Table S1. True positive (TP), true negative (TN), false positive (FP), and false negative (FN) cases for the three anatomical sites of the study regarding cytological diagnosis, using the histological result as the gold standard.

	TP	TN	FP	FN	Total
**FALLOPIAN TUBES**	22	434	21	14	491
**OVARIES**	23	401	20	47	491
**ADNEXA**	25	217	43	5	290

Table S2. Performance indicators for each anatomical site.

	FALLOPIAN TUBES (%, 95% CI)	OVARIES (%, 95% CI)	ADNEXA (%, 95% CI)
**Sensitivity**	61.1% (45% – 77.1%)	32.9% (20.3% – 45.5%)	36.8% (25.4% – 49.3%)
**Specificity**	95.4% (92.8% – 97.6%)	95.2% (92.5% – 97.4%)	97.7% (94.8% – 99.3%)
**PPV**	51.2% (32.6% – 70%)	53.5% (34.8% – 71.1%)	83.3% (65.3% – 94.4%)
**NPV**	96.9% (94.9% – 98.5%)	89.5% (86.2% – 92.6%)	83.5% (78.4% – 87.8%)
**FPR**	4.6% (2.4% – 7.2%)	4.8% (2.6% – 7.5%)	2.3% (0.7% – 5.2%)
**FNR**	38.9% (22.9% – 55%)	67.1% (54.5% – 79.7%)	63.2% (50.7% – 74.6%)
**OA**	92.9% (90% – 95.3%)	86.4% (82.4% – 89.7%)	83.4% (78.7% – 87.5%)
**PLR**	13.2 (7.6 – 25.7)	6.9 (3.6 – 13.3%)	16.3 (6.5 – 41)
**NLR**	0.4 (0.2 – 0.6)	0.7 (0.6 – 0.8%)	0.6 (0.5 – 0.8)
**Diagnostic Odds Ratio**	32.5 (14 – 86.5)	9.8 (4.4 – 22%)	25.2 (9.1 – 69.6)
**Youden Index**	56.5% (39.8% – 72.7%)	28.1% (14.8% – 40.6%)	34.5% (22.9% – 46.1%)
